# Laminar Biomaterial Composite of PVA Cryogel with Amnion as Potential Wound Dressing

**DOI:** 10.3390/polym15132955

**Published:** 2023-07-05

**Authors:** Łukasz Otulakowski, Agnieszka Klama-Baryła, Anna Celny, Maciej Kasprów, Anna Hercog, Marcin Godzierz, Anna Sitkowska, Sławomir Kadłubowski, Magdalena Jaworska, Ewa Chmielik, Barbara Trzebicka, Alicja Utrata-Wesołek

**Affiliations:** 1 Centre of Polymer and Carbon Materials, Polish Academy of Sciences, M. Curie-Skłodowskiej 34, 41-819 Zabrze, Poland; acelny@cmpw-pan.pl (A.C.); mkasprow@cmpw-pan.pl (M.K.); ahercog@cmpw-pan.pl (A.H.); mgodzierz@cmpw-pan.pl (M.G.); btrzebicka@cmpw-pan.pl (B.T.); 2 Dr. Stanislaw Sakiel Center for Burn Treatment, 2 Jana Pawla II St., 41-100 Siemianowice Śląskie, Poland; aklama@clo.com.pl (A.K.-B.); asitkowska@clo.com.pl (A.S.); 3 Institute of Applied Radiation Chemistry, Chemistry Faculty, Lodz University of Technology, Wróblewskiego 15, 90-924 Łódź, Poland; slawomir.kadlubowski@p.lodz.pl; 4 Tumor Pathology Department, Maria Skłodowska-Curie National Research Institute of Oncology Gliwice Branch, Wybrzeże Armii Krajowej 15, 44-102 Gliwice, Poland; magdalena.jaworska@io.gliwice.pl (M.J.); ewa.chmielik@io.gliwice.pl (E.C.)

**Keywords:** poly(vinyl alcohol), cryogel, amnion, wound dressing

## Abstract

Gel dressings, composed of polymers both natural and synthetic, are successfully used in the treatment of burn wounds. They protect the burn wound site against adverse external factors, ensure an adequate level of tissue hydration, have soothing and pain-relieving properties, and also support the healing process and reduce the risk of pathological scars. Another promising material that can be used in the wound-healing process is an amnion membrane. Due to its valuable properties such as protecting the body against bacterial infections and permeability to nutrition, it has found usage in different brands of medicine. In this work, we have combined the beneficial properties of hydrogels and amnion in order to make the laminar dressing that may serve for wound healing. For that purpose, the physically crosslinked cryogel of poly(vinyl alcohol) (PVA) was covered with an amnion membrane. Subsequently, gamma irradiation was performed, leading to the simultaneous internal crosslinking of the hydrogel, its permanent bonding with the amnion, and dressing sterilization. The physicochemical properties of the dressing including gel fraction, swelling, and hardness were studied. Biological tests such as the MTT assay, antimicrobial activity, and histopathological examination confirmed that the obtained material constituted a promising candidate for further, more in-depth studies aiming at wound dressing application.

## 1. Introduction

In medicine, in the case of treating patients with minor skin damage, the primary medical intervention is the application of a dressing. The use of dressing with optimal properties is a key aspect of the healing process of the damaged area. Traditional dressings used in medicine for a number of years include plasters, bandages, and gauze. The role of these measures is to protect the wound against contamination by impurities, further mechanical damage, and irritation. Unfortunately, traditionally used dressings possess numerous disadvantages, one of the biggest concerns the growth of the non-impregnated fibers into a wound and excessive drying of the wound due to the high gas permeability of the dressing. In other cases, insufficient absorption of secretions can also be problematic. Currently, supporting the healing process and minimizing complications is a basic role of modern wound dressings. J. Dissemond et al. [1] well summarized the properties that a modern dressing should possess. Besides obviously being non-toxic, the dressing should create a sterile environment and a barrier to infection. It should also provide high humidity for the wound environment and help to support optimal temperature and gas permeation. The epithelialization, angiogenesis, and regeneration of connective tissue should not be disturbed while using the dressing. Additionally, as mechanical properties are also crucial, the dressing material should allow for easy cutting to the desired size and easy shaping during placement on the wound.

Materials that show most of these properties are hydrogels. Hydrogels are highly hydrophilic macromolecular networks which are acquired by chemical or physical crosslinking of polymer chains in solution. Hydrogel polymer networks may absorb high quantities of water up to thousands of times their dry weight [2]; the water content in hydrogels is similar to that in human tissue. Due to that, hydrogels have been of great interest to biomaterial scientists for many years [3,4,5]. Hydrogels are usually stabilized by the formation of crosslinking points between polymer chains by chemical linkages or physical interactions. Physical- or radiation-crosslinked hydrogels are preferred over those formed by chemical crosslinking as a large number of catalysts, crosslinkers, or other additives that can present some level of toxicity are avoided [6]. The most important advantages of hydrogels used in wound dressings are their easy preparation and painless application to the wound. Hydrogel-based dressings are present for commercial usage under various names such as Aquasorb, Neoheal, Granuflex, Nu-Gel, AquaGel, and others prepared by different manufacturers. For the preparation of hydrogel wound dressing, natural polymers, such as polysaccharides or proteins, and synthetic polymers, such as poly(vinylpyrrolidone), poly(ethylene glycol), or poly(vinyl alcohol), are extensively used [7]. Poly(vinyl alcohol) (PVA) is a highly biocompatible, non-toxic, non-carcinogenic, and hydrophilic polymer. It provides excellent water absorption, mucoadhesion, and oxygen permeability, and also it ensures good chemical/enzymatic resistance [8]. The preparation of PVA-based hydrogels is developed through crosslinking techniques that can be categorized as physical and chemical crosslinking [9]. Physical crosslinking involves the formation of crystallites as crosslinking points and can be achieved by using repeated freezing and thawing, and so-called cryogels are formed [10]. This method of obtaining PVA gels is advantageous over chemical crosslinking due to its simplicity and lack of additives, such as crosslinkers or photosensitizers that can be harmful to cells or cause inflammation. Cryogels also exhibit great porosity and their physical properties can be easily tailored [10]. Chemical crosslinking, involving the formation of covalent bonds as crosslinking points, uses chemical agents as crosslinkers or irradiative techniques such as electron beams or gamma radiation. Further, the radiation technique can be combined with the physical crosslinking technique for the synthesis of PVA hydrogels with improved properties [11,12,13]. The PVA can be processed not only as a hydrogel dressing form but also as foams, films, particles, sponges, and fibers, where it is usually blended with other polymers (mainly naturally based polymers) [6].

The main advantage of hydrogels is the possibility to incorporate active substances, e.g., antibacterial or antiseptic agents within the dressing, which can additionally support the healing of the wound [14,15]. A substance that can be added to wound dressing to promote healing is one of the fetal membranes, namely the amnion membrane. The amniotic membrane surrounds the embryo of birds, reptiles, and mammals, including humans [16]. A diagram presenting placement of amnion in human body and its detailed structure can be found in [17]. It is thin, semi-permeable tissue consisting mostly of collagen that makes up the innermost layer of the fetal bladder. The extracted amnion membrane consists of five layers (1) epithelium layer, (2) basement membrane, (3) compact layer, (4) fibroblast layer, and (5) spongy layer [18,19]. The amniotic membrane also contains growth factors, cytokines, and signaling molecules [20,21]. In the case of a fetus, the amnion provides a suitable environment and protects the embryo from mechanical damage. The amnion itself can be used for the healing of wounds due to its numerous advantageous properties. It is known that amnion accelerates healing and can reduce the loss of proteins, fluids, and electrolytes, reducing the soreness of the wounded area. Since the amniotic membrane is rich in nutrients, has low immunogenicity and the ability to inhibit the growth of various microorganisms [22,23,24], it is often used as a dressing on its own in the treatment of skin lesions [25,26,27]. It has also been shown that the amnion possesses both angiogenic and anti-angiogenic properties depending on which side of the amnion is considered. The anti-angiogenic property of the amnion belongs to the amniotic epithelial cells, while the mesenchymal side of the amnion can promote angiogenesis by inducing the proliferation of endothelial cells [18]. Amnion membranes have been successfully applied in medical use for wound and reconstructive purposes since the early 20th century. The wound healing process with amnion is presented, for example, in [20], and the mechanism of its action has been proposed [28,29]. In 1910, Davis was the first to report the use of amnion membranes as surgical material in skin transplant procedures [30]. Since these early reports identified the amnion membrane as a material with much potential in wound healing, the amnion membrane has become relatively widespread in many applications connected to wounds, from a simple covering material through the healing of ocular surface disorders, diabetic neurovascular ulcers, and venous stasis ulcers to various types of post-surgical and post-traumatic wound dehiscence [31,32,33].

In this work, we describe a novel approach for the preparation of wound dressings with an amnion monolayer on the surface of hydrogel to combine the positive influence of the hydrogel and amnion membrane. In the presented methodology, the pre-prepared physical cryogel of poly(vinyl alcohol) was covered with an amnion membrane. Subsequently, gamma irradiation was performed, which led to the simultaneous internal crosslinking of the hydrogel, its permanent bonding with the amnion membrane layer, and dressing sterilization. The idea is that the amnion layer with its healing properties will directly adhere to the wound, and the hydrogel layer will provide a proper moisturizing environment and the ability to adsorb the antiseptic or antibacterial compounds. An additional advantage of the obtained laminar wound dressing is the fact that the procedure of its preparation takes place simultaneously with its sterilization. In the article, the route for obtaining the dressing is depicted as well as its biological properties such as cytotoxicity, antimicrobial activity, and histopathological examination.

## 2. Materials and Methods

### 2.1. Materials

Poly(vinyl alcohol) (PVA) “Mowiol^®^” (Mw = 195 kDa; 98.0–98.8 mol% of hydrolysis) was purchased from Sigma-Aldrich (Taufkirchen, Germany) and used as received. Glutaraldehyde (GA) 50% in water solution was purchased from Fluka (Buchs, Switzerland) and used as received. The water used for the preparation of PVA solutions was purified using a commercial ion exchange system (Hydrolab, Straszyn, Poland) and filtered through a 0.2 µm filter (Millipore; Burlington, MA, USA). Materials for biological testing such as Sutrisept antiseptic (ACTO GmbH, Braunschweig, Germany), CyQUANTTMMTT Cell Viability Assay Kit (Life Technologies Corporation, Eugene, OR, USA), Dulbecco’s Modified Eagle’s Medium (DMEM, Mediatech Inc., Manassas, VA, USA), FBS (Mediatech Inc., Woodland, CA, USA), Gentamicin (Grand Island, NY, USA), Amphotericin B (Life Technologies Limited, Paisley, UK), PBS (Mediatech Inc., Manassas, VA, USA), DMSO (WAK-Chemie Medical GmbH, Steinbach, Germany), and TaliTM Apoptosis Kit (Life Technologies Corporation, Eugene, OR, USA) were used as received.

Porcine placentas were collected during natural births under aseptic conditions. Pigs from the MEDPIG project (license no INNOMED/I/17/NCBR/2014) were bred in the Experimental Department of the National Research Institute of Animal Production in Żerniki Wielkie, Poland. Each obtained placenta was rinsed with the saline solution. Then, the amnion was placed under the laminar flow cabinet, manually separated from the chorionic layer, and again rinsed with the saline solution. The amnion was cleaned from unnecessary biological waste. The amnion was then cut into circles of 5 cm in diameter. Human dermal fibroblasts were derived from the patient and stored in the Tissue Bank at the Dr. Stanislaw Sakiel Center for Burns Treatment in Siemianowice Śląskie. Bacteria strains for antibacterial studies were *Escherichia coli* ATCC 25922, and *Staphylococcus aureus* ATCC 29213.

### 2.2. Methods for Preparation of the Dressing

The preparation of the polymer solutions and further the dressing was performed in a laminar chamber to avoid the contamination.

#### 2.2.1. PVA Cryogel Preparation

PVA solutions of 5 and 10 wt% were prepared by direct dissolution of polymer in ultrapure water. First, the polymer was placed in water for 24 h to swell. Next, the polymer was dissolved by increasing the temperature to 90 °C with constant mixing and incubated for 24 h to ensure complete dissolution. The container was kept sealed during the process to avoid drying. A clear viscous solution was acquired. The solution was cooled and stored at room temperature. Next, 5 mL of polymer solution was slowly poured into Petri dishes (5 cm in diameter) and closed.

PVA cryogel samples were obtained by subjecting the polymer aqueous solutions to four freezing–thawing cycles according to procedure described in [13,34]. Each freezing cycle lasted for 4 h at −4 °C, after which gel was allowed to warm up to room temperature for an hour. When completed, the cryogels were stored in tightly sealed Petri dishes in the fridge (+4 °C) prior to use.

#### 2.2.2. Dressing Preparation

Hardened PVA cryogels were coated with a small amount (0.1 mL) of 10% or 50% glutaraldehyde water solution. Immediately after that, a circle of amnion membrane (5 cm in diameter) was placed gently on the gel. With the help of tweezers, the amnion was carefully spread, taking care that there were no air bubbles. Each Petri dish with dressing was sealed with parafilm, vacuum-packed in sterilization bags, and submitted to e-beam irradiation. The glutaraldehyde was in contact with the amnion and PVA cryogels for about 24 h. After radiation crosslinking, the residues of glutaraldehyde were removed by washing the dressing in 10 mL of clean deionized water 5 times for 5 min each.

#### 2.2.3. E-Beam Irradiation

Each of separately vacuum-packed dressing samples was irradiated with the 25 kGy and 35 kGy doses of accelerated electron beam radiation. The purpose of this step was to perform radiation-induced crosslinking between PVA and amnion simultaneously with sterilization of the dressing. All the tested samples were irradiated using a high-speed electron beam generated by the ELU6e linear accelerator (Elektronika, Moscow, Russia). The dishes were placed perpendicular to the axis of the electron beam at a fixed distance of 160 cm from the beam exit window. A beam of 6 MeV electrons, emitted in pulses of 4 µs duration at 20 Hz, was used to irradiate the samples. Irradiations were performed at room temperature in the presence of air. The dose rate (according to alanine dosimetry) was 4.5 kGy/min.

### 2.3. Characterization of Dressing Properties

#### 2.3.1. Measurements of Gel Fraction Yield and Equilibrium Degree of Swelling

Gel fraction (*GF*) yield and swelling degree (*SD*) of the PVA cryogels before and after irradiation were determined gravimetrically. Gel samples of dimensions 10 mm× 10 mm were cut from cryogel disc for measurements.

For GF calculation, the samples were dried to a constant weight, transferred to conical flasks, flooded with 100 mL of deionized water and left on a shaker at 40 or 90 °C for 24 h. After this time, the swollen hydrogels were withdrawn from the solution, gently dried with Kimtech dust-free wipes, and dried to a constant weight. For verification, the entire procedure was repeated. The gel fraction was calculated according to the formula:

$$GF=\frac{m_{x}}{m_{y}}\cdot 100\%$$

where:

*m_x_*—weight of the dried cryogel after extraction

*m_y_*—initial weight of the dried cryogel

Prior to *SD* measurements, the gel samples were extracted in distilled water for 24 h at room temperature, dried, and weighed. After that, the gels were placed in a 20 mL vial and poured with 10 mL of deionized water at room temperature. For a period of 2 h, every 20 min, a cryogel sample was taken out and dried with Kimtech dust-free wipes, then weighed. After this time, the samples were weighed 5 more times in a 24-h period. Values for the degree of swelling of the sample were calculated from the formula:

$$SD=\frac{m_{hydr}-m_{s}}{m_{s}}\cdot 100\%$$

*m_hydr_*—weight of hydrated samples

*m_s_*—weight of dried sample

The reported results are the average of measurements performed for at last two samples each.

#### 2.3.2. Mechanical Properties

The hardness of the dressings was determined using a Shore durometer on the A scale. The results were recalculated to present the Young modulus. Measurements were performed for 3 samples on 5 points on the whole surface of the polymeric gel. Using linear elastic indentation hardness, a relationship between the ASTM D2240 hardness (*S*) and Young’s modulus (*E*) for elastomers was derived by Gent and by Mix and Alan Jeffrey Giacomin. Gent’s relation has the form [35]:

$$E=\frac{0.0981\left(56+7.62336S\right)}{0.137505\left(254-2.54S\right)}$$

#### 2.3.3. Scanning Electron Microscopy

The microstructures of the sample were characterized using a scanning electron microscope (SEM, FEI Company, Brno, Czech Republic, Quanta 250 FEG). Two different SEM techniques were applied: low vacuum (LV) and environmental conditions (ESEM). For the LV measurements, samples were immersed in liquid nitrogen and lyophilized for 24 h under low pressure (Alpha 1-2LDplus, Christ, Osterode am Harz, Germany). The microphotographs of the cross-section were obtained under a low vacuum (80 Pa) with an acceleration voltage of 5 kV from secondary electrons collected by a Large Field Detector (LFD) (FEI Company, Brno, Czech Republic). The ESEM micrographs of the surface were obtained under the ESEM mode (5.4 Torr, 2 °C) using the Peltier Stage, with an acceleration voltage of 10 kV and 15 kV from secondary electrons collected by a Gaseous Secondary Electron Detector (GSED) (FEI Company, Brno, Czech Republic). The samples were kept wet all the time during the analysis.

#### 2.3.4. X-ray Studies

X-ray diffraction (XRD) was performed using the D8 Advance diffractometer (Bruker, Karlsruhe, Germany) with Cu-Kα cathode (λ = 1.54 Å). The scan rate was 0.27°/min with scanning step 0.02° in a range of 15° to 35° 2Θ (dwell time 4 s). The 2Θ range was chosen based on Ricciardi and Holloway [36,37], which indicated that the primary crystalline peak for PVA occurs at 19.4 2Θ. This corresponds to a d-spacing of 4.68 Å and crystalline dimensions in the (1-0-1) lattice direction. All measurements were acquired at least two times and summed, then the obtained profiles were smoothed using a 15-point, quadratic polynomial Savitzky–Golay smoothing filter. The crystallinity level of hydrogels was calculated using the peak decomposition method, according to the equation:

Relative crystallinity (%) = (PVA crystalline peak area)/(total area) × 100%

where the PVA crystalline peak area is the area at 19.4 2Θ and the total area is the entire area within the 2Θ range of 15–35. The obtained crystallinity represents a relative value and can be compared with that of other samples, provided the same testing method is used; however, it does not represent an exact measurement of the crystallinity percentage as the equation is not normalized using known crystallinity values.

### 2.4. Biological Studies

#### 2.4.1. Cytotoxicity Assay of Extract of PVA/Amnion Material

The MTT assay was applied to follow the cytotoxicity of the extract obtained from amnion/PVA laminar material, according to the procedure described in [38,39]. Briefly, the samples of crosslinked cryogels of PVA/amnion were immersed in the growth medium DMEM with 10% FBS and Antibiotic-Antimycotic and placed at 37 °C for 24 h to produce extraction media. Simultaneously, human fibroblasts were seeded in a 96-well tissue culture microplate at a concentration of 7000 cells per well, and incubated in 200 µL of DMEM growth medium at 37 °C and c(CO_2_) = 5% for 24 h. After this time, the growth medium was replaced with a 200 μL of extraction medium, and the cells were incubated (at 37 °C and 5% CO_2_) for an additional 24, 48, and 72 h. Control and test samples were performed in triplicate. For the MTT test, a 12 mM MTT (3-(4,5-dimethylthiazol-2-yl)-2,5-diphenyltetrazolium bromide) stock solution was prepared in PBS. To detect formazan, a product of the redox reaction of viable cells, a rapid protocol using DMSO as a solubilizing agent was used. After a given culture time, the standard 200 µL of extraction medium was replaced with 100 µL DMEM, and then the 12 mM MTT stock solution was added to each well and incubated at 37 °C for 4 h. After 4 h, DMSO solution was added to each well, and the microplate was incubated at 37 °C for 10 min. The absorbance of the solution was measured at 540 nm using a Multiskan Sky Microplate Spectrophotometer (Thermo Fisher Scientific, Göteborg, Sweden).

#### 2.4.2. Cell Proliferation and Death Studies

For evaluation of the proliferation and apoptosis of the cells, the samples of PVA/amnion were immersed in DMEM with 10% FBS and Antibiotic-Antimycotic solution and placed at 37 °C for 24 h to produce extraction media as was described previously [38,39]. Simultaneously, human fibroblasts were seeded 1 × 10^5^ per well. Cells were grown for 24 h. After this time, in half of the wells, the growth medium was replaced with an extraction medium, and in the rest of the wells, the growth medium was replaced with fresh DMEM. Cells were grown in DMEM or an extraction medium for 24 h and 72 h. After an appropriate time, the cells were detached with trypsin solution. Tali^®^ Viability and Apoptosis Tests were performed on the solution of the trypsinized cells. Briefly, the harvested cells were centrifuged, and the supernatant was discarded. The pellet of the cells was resuspended in 100 µL of 1× Annexin Binding Buffer. Annexin V Alexa Fluor^®^488 and, subsequently, Tali^®^ Propidium Iodide was added to the solution. Annexin V Alexa Fluor^®^488 can identify apoptotic cells by binding to phosphatidyl serine exposed on the outer leaflet. Propidium Iodide is a DNA-binding dye which is used for identifying necrotic cells. After incubation, the solution of cells was loaded into a Tali^®^ Cellular Analysis Slide, and a probe was measured in a Tali Imaged-Based Cytometer (Life Technologies Corporation, Carlsbad, CA, USA).

#### 2.4.3. Antibacterial Studies

The disk diffusion test was used to investigate the antibacterial activity of the amnion/PVA laminar wound dressing against *Staphylococcus aureus* and *Escherichia coli* in accordance with literature [40]. To evaluate the antibacterial activity, a standard zone of inhibition test was performed. PVA discs, PVA + amnion discs, and PVA + amnion + antiseptic discs (soaked in Sutrisept antiseptic for 3 h) were prepared. The discs were applied aseptically to the surface of the inoculated plates at an appropriate special arrangement (3 discs per one bacteria strain) and incubated at 37 °C for 24 h. Then, the plates were examined, the diameters of the zone of inhibition were observed, and the inhibition of bacterial growth was visually evaluated.

#### 2.4.4. Histology of the Amnion and Its Laminar Dressing with Cryogel

All processed samples of amnion and laminar dressing were stored in 10% buffered formaldehyde at room temperature until the histological slides were prepared. The preparations were stained in a standard way with hematoxylin and eosin (H&E). The evaluation of the preparations was carried out on an Olympus BX43 microscope (Evident Europe GmbH, Hamburg, Germany).

## 3. Results and Discussion

**Scheme 1:**
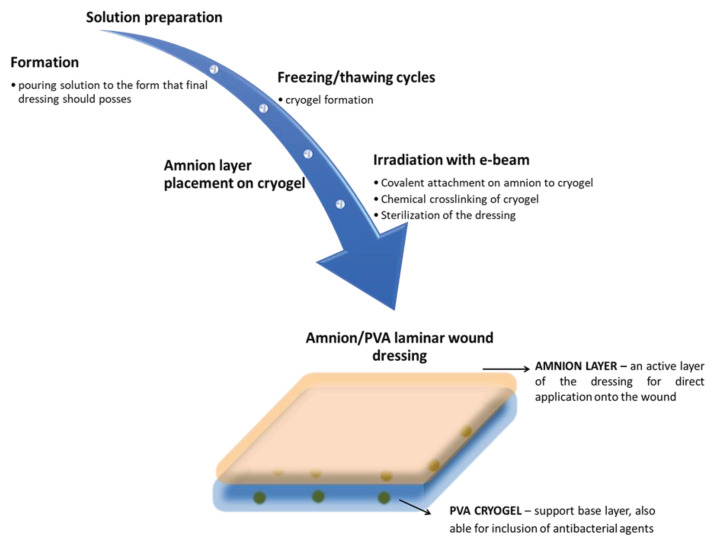
A schematic representation of a preparation procedure of a laminar wound dressing based on PVA and amnion.

The hydrogel wound dressing was prepared in two steps. The first step was to acquire a cryogel from a PVA solution by cyclic freezing and thawing of samples in the refrigerator. This process allowed to achieve firm, physically crosslinked gel due to the formation of structured crystalline domains of the polymer chains through phase separation acting as crosslinking points [4,41]. The cryogel layer served as a support for the amnion layer, which was laminated on the top of the cryogel from the epithelial cell side. Such an arrangement of the amnion layer will result in a dressing that will rest on a wound from the mesenchymal side. This should lead to the clonogenicity and differentiation potency of the dressing, providing its anti-inflammatory and angiogenetic properties [19] simultaneously, which, finally, should accelerate the wound healing process. Such a prepared dressing of cryogel with amnion was irradiated with an e-beam. This step, due to the formation of radicals, ensured a covalent connection between the hydrogel and amnion layer and also covalent crosslinking within the gel matrix. An advantage is that the irradiation was carried out in a sterilization bag, so at the same time, it was possible to obtain sterile dressings. The procedure for the preparation of the dressing is presented in Scheme 1.

### 3.1. Cryogel Formation and Characterization

Before the formation of the dressing with the amnion layer, the cryogels from PVA were prepared and characterized in order to check whether the physicochemical properties of the obtained gel, serving as a base material for the amnion, would be appropriate to act as a dressing.

The cryogels were prepared from the 5 wt% and 10 wt% aqueous solutions. Four cycles of freezing–thawing (FTC) were performed. The structure and properties of a cryogel formed by a freeze–thaw cycle are influenced by polymer molar mass and its solution concentration [42], conditions of the FTCs, such as freezing and thawing rate, number of cycles, and temperatures at each stage [43]. It was shown in [37] that the PVA cryogels with good mechanical properties are formed already after three freezing–thawing cycles, and further cycles do not significantly influence the degree of PVA crystallization and the final properties of the cryogels. Based on that, four cycles of freezing–thawing were chosen in our case to prepare cryogels. The gels were irradiated with an e-beam with a dose of 25 and 35 kGy. The gel fraction (*GF*) and swelling degree (*SD*) values for samples before e-beam irradiation (PVA_NOT-IRR_) and after e-beam irradiation (PVA_IRR_) are summarized in Table 1.

PVA cryogels, regardless if they were irradiated or not, were highly crosslinked as the obtained *GF* values were in the range of 81–96%. After additional washing of the samples, the change in the *GF* value was negligible (data not shown).

The cryogels obtained without e-beam irradiation (PVA_NOT-IRR_) were stable at temperatures up to 40 °C. Washing at a higher temperature (90 °C) caused the disintegration of the gel structure due to the dissolution of crystalline domains that acted as crosslinking points.

Cryogels of PVA were subjected to e-beam irradiation (PVA_IRR_). During irradiation, the radiolysis of water by fast electrons is performed and leads to an early homogeneously distributed reactive species, the most important being hydroxyl radicals, hydrogen atoms, and hydrated electrons [44]. These species are capable of abstracting hydrogen atoms from polymer chains in very fast reactions, which leads to the formation of radicals randomly distributed along the polymer chain. The recombination between two radicals results in the formation of new C-C bonds and, thus, crosslinking [45].

The PVA_IRR_ cryogels remained stable even at 90 °C, the temperature at which they were washed (Table 1). This means that although the physical crosslinking points are disintegrated at this temperature, the irradiation led to chemical crosslinking and stable cryogels. However, the *GF* of PVA_IRR_ was lower at 90 °C as compared to samples washed at 40 °C, meaning that some of the polymer chains were not crosslinked.

**Figure 1:**
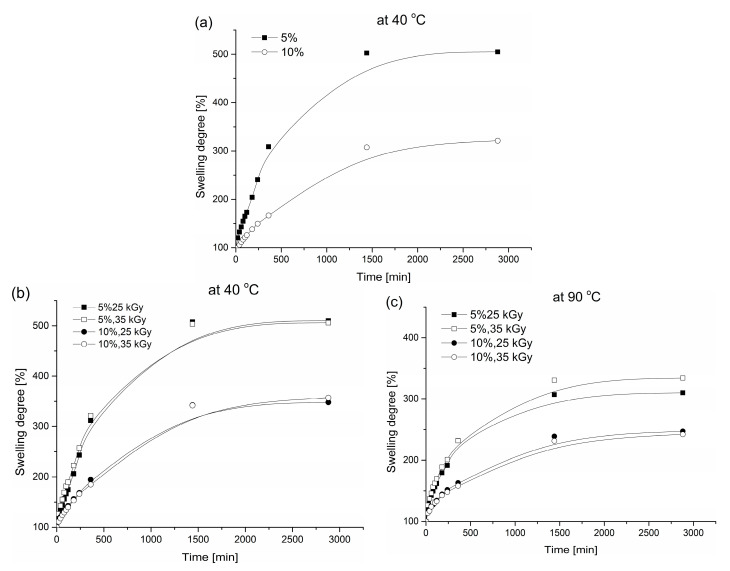
Swelling degree of PVA cryogel: (**a**) PVA_NOT-IRR_ washed at 40 °C, (**b**) PVA_IRR_ washed at 40 °C, and (**c**) PVA_IRR_ washed at 90 °C.

The higher radiation dose resulted in a slightly higher gel fraction of the samples. The influence of the concentration of PVA on *GF* can be noticed but is not significant. Similar to the gel fraction, the swelling degree of gels was studied for samples before (Figure 1a) and after irradiation (Figure 1b,c).

All the acquired gels swelled fast during the first 6 h of incubation. After that, during the next 24 h, the swelling rate lowered. Initial fast swelling is common for gels with homogenously distributed pores [46]. The studied samples reached the equilibrium *SD* over approximately 40 h, and afterwards, the cryogels did not absorb significant volumes of water. The equilibrium *SD* varied between 195 and 440%. As can be seen in Figure 1 and Table 1, a more concentrated solution used for the preparation of gels led to a lower *SD* of the resultant cryogels (both for PVA_NOT-IRR_ and PVA_IRR_ gels). This indicates that the crosslinking degree for the samples obtained from the more concentrated polymer is higher, and a higher number of polymer chains are available for network formation. A decrease in the equilibrium *SD* can also be observed for the PVA_IRR_ samples washed at 90 °C in comparison to samples washed at 40 °C, indicating the higher degree of crosslinking of the first.

The relative crystallinity of PVA cryogels was determined by the XRD technique (Table 2).

As can be seen in Table 2, a slight decrease in crystallinity appeared for PVA_IRR_. Similar dependence and a reduced fraction in crystallinity of the freeze–thaw cryogels after irradiation was observed for poly(vinyl alcohol) [12] and PVA/PEG hybrid [13] samples. A much greater degree of crystallinity was achieved for samples obtained from higher concentrated PVA solutions due to the higher fraction of polymer-rich regions able to crystalize [37]. The crystallite size of PVA crystalline fraction, calculated by the Scherrer equation, is in the range of 8 to 11 nm for both the non-irradiated and irradiated cryogels.

**Figure 2:**
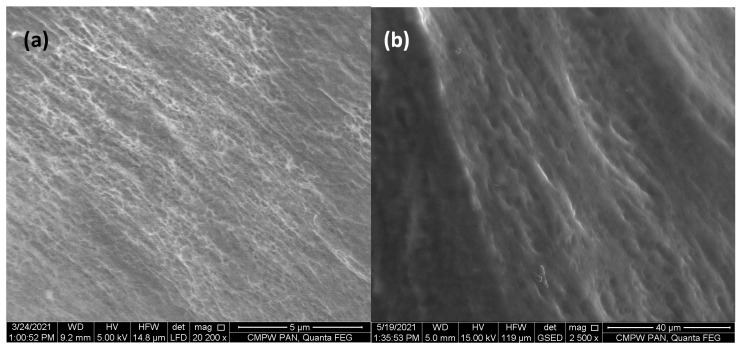
SEM images of fractures morphologies of exemplifying PVA_IRR_ (10%, 35 kGy) gel: (**a**) lyophilized and (**b**) hydrated.

Surface morphologies of the PVA cryogels were investigated using SEM. Two modes were applied: standard scanning electron microscopy after lyophilization of cryogels and when the gels were hydrated (in environmental conditions ESEM). The obtained images are presented in Figure 2.

Cryogels have microporous structures and interconnected flow channels. In the lyophilized sample, the pore size reached about 300 nm. The pore size in hydrated gels varied between 2 and 8 µm. When the cryogel is hydrated, the walls of the pores are smooth. During the polymer swelling, the pores are filled with water; thus, their sizes increase significantly.

Hydrogels and dressings made in this study were designed to be part of soft matter materials. Mechanical properties of wound dressing materials should ensure that they will adapt to the wound bed topography and contour, will not disintegrate, and will not cause discomfort (for example, due to their stiffness) during usage. PVA is an attractive candidate for many applications in regenerative tissue medicine as it can mimic a wide range of soft tissues [43,47,48,49,50]. The hardness of the PVA_IRR_ gels obtained in this work was measured using a Shore durometer on the A scale (Table 3). Gels before e-beam treatment were too soft to measure them reliably; thus, these results are not presented.

Mechanical properties of PVA cryogels depend on, among others, the number of freezing–thawing cycles and the thaw and freeze rate [51,52]. The Young modulus of PVA cryogels can vary from around 20 kPa to over 300 MPa [52,53]. For the PVA_IRR_ cryogels studied here, the calculated values of the Young modulus were in the range of 160 to 266 kPa. After the e-beam, the crystallinity of the samples decreases in comparison to non-irradiated samples, and the crystallite size is the same, suggesting the formation of covalent bonds due to chemical crosslinking instead of crystal growth. Thus, this process leads to an increase in the overall hardness of the samples. According to expectations, the values of the Young modulus increased with the increasing concentration of polymer used for its preparation. The more concentrated gel had improved mechanical properties due to increased sample crystallinity. Gels formed from solution at 5 wt% were delicate, fragile, and difficult to handle. They could be damaged very easily, even after irradiation. Gels made from a 10 wt% solution after irradiation had higher mechanical strength compared to 5 wt%, regardless of the radiation dose. They were very flexible and soft but easy to handle.

Our studies showed that the most favorable properties for the application as the wound dressing possessed cryogel composed of 10 wt% PVA. The material was soft but still stiff enough for easy handling. Its properties allowed for easy cutting to the desired size and fitting to the desired shape. Moreover, the 10 wt% gel did not shrink under e-beam influence, which is important during the stage of amnion application. The Young modulus for 10 wt% gels irradiated with 35 kGy is only slightly higher than for gels irradiated with 25 kGy. However, these gels were still elastic and firm and did not damage when pulled. Additionally, according to the literature [54], the irradiation of human allogeneic skin grafts with a dose of about 35 kGy, although more expensive, possesses the highest decontaminating values against microorganisms and viruses. That is why the 35 kGy dose was chosen for procedures of amnion attachment to the PVA cryogel with simultaneous sterilization of the dressing.

### 3.2. Preparation of the Dressing

**Figure 3:**
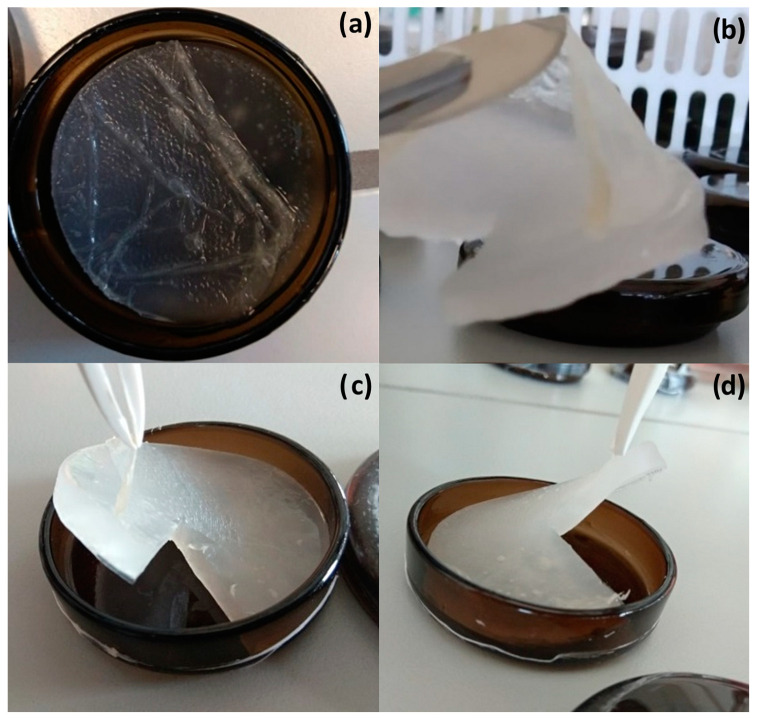
Pictures of the amnion on the cryogel: (**a**) irradiated without any additives between the amnion and cryogel, (**b**) with glutaraldehyde solution between layers of amnion and cryogel but no irradiation, (**c**) irradiated with the use of 10% glutaraldehyde solution between the amnion and cryogel, and (**d**) irradiated with the use of 50% glutaraldehyde solution between the amnion and cryogel.

The schematic procedure for the formation of the laminar wound dressing is presented in Scheme 1. An amnion with a diameter of 5 cm was placed on a 5 cm circle of the cryogel, obtained from 10 wt% PVA solution after 4 cycles of freezing–thawing. In addition, a small amount of 10 or 50% glutaraldehyde water solution was also applied between the layers of amnion and cryogel. In combination with e-beam irradiation, this was necessary to ensure stable membrane immobilization on the surface of the gel. Without glutaraldehyde, the irradiation alone resulted in not sufficient attachment of the amnion over the whole cryogel, the amnion partially slipped over the cryogel and was only attached to the gel in some places (Figure 3a).

The combination of the usage of a small amount of GA (0.1 mL on whole surface of the sample, ~20 cm^2^) and irradiation resulted in a strong connection of the amnion with cryogel and formation of laminar dressing (Figure 3c,d). The best connection of the amnion with cryogel was obtained when 50% solution of glutaraldehyde was used. For comparison purposes, when glutaraldehyde was only used (incubation period 24 h), without e-beam irradiation of the dressing, the connection of the amnion layer with the PVA cryogel was visible only on the edges of the gel (Figure 3b). This confirms that the irradiation is a crucial factor for proper connection of the amnion with cryogel.

**Figure 4:**
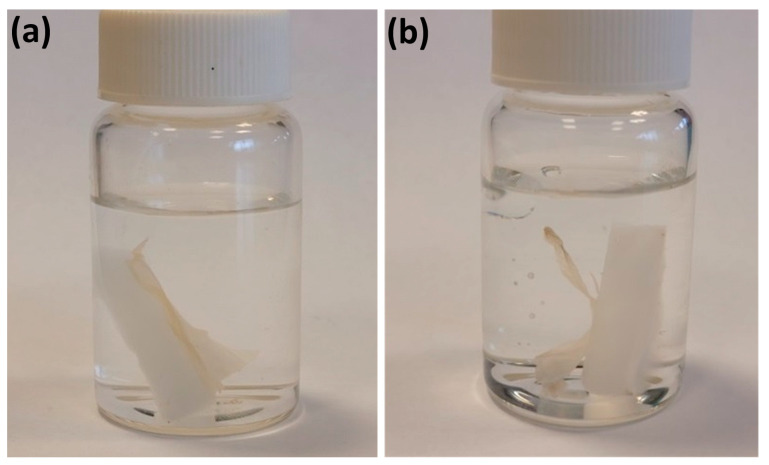
A picture of the laminar dressing immersed in water for 24 h: (**a**) samples with 50% of GA and irradiation applied, (**b**) samples with 10% of GA and irradiation applied.

The stable attachment of the amnion to the cryogel was evidenced by immersing the dressings in water (Figure 4). It can be seen that when a 50% solution of glutaraldehyde and irradiation was applied, after 24 h of immersion, the amnion still stuck on the cryogel (Figure 4a). Only some slight detachment of the amnion from the edges of the cryogel was noticed. The dressing, after immersion in water, was stable for at least 5 days without visible disintegration of the gel itself or the removal of the amnion from its surface. On the other hand, the use of 10% glutaraldehyde solution and electron beam irradiation resulted in a dressing in which the amnion detached to a larger extent after being submerged in water, although some parts of the amnion were still bound (Figure 4b). This shows that only a formulation with highly concentrated glutaraldehyde is suitable for dressing preparation, providing a stable connection between the amnion and polymer cryogel.

**Figure 5:**
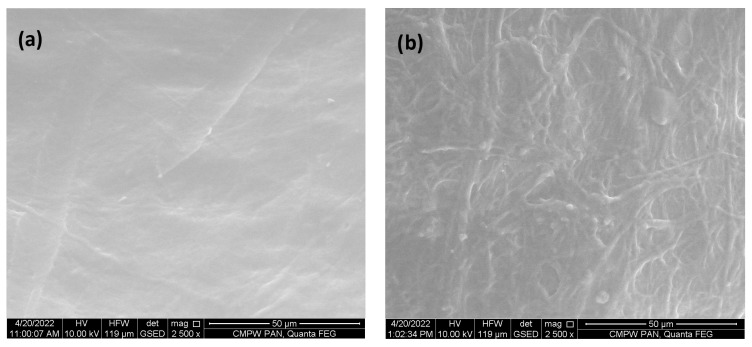
SEM morphologies of the (**a**) PVA_IRR_ (10%, 35 kGy) cryogel surface and (**b**) surface of the laminar dressing with visible amnion.

The SEM analysis also confirmed the presence of the amnion attached to the cryogel (Figure 5). The surface of the pure cryogel is smooth (Figure 5a), while the network of collagen fibers derived from the amnion on the surface of the dressing is well visible (Figure 5b).

### 3.3. Biological Studies

The hydrogel laminar dressing, sterile packed after irradiation, was then used for biological research. After unpacking, the dressing was first washed and then subjected to a MTT test to verify its cytotoxicity. The test of proliferation and apoptosis was also performed. Antibacterial studies gave information on the ability of the material to inhibit bacterial growth, while histological examinations concerned the maintenance of histologically normal architecture of the amnion.

#### 3.3.1. Cytotoxicity Studies of the Extract from Amnion/PVA Material via MTT Test

The MTT assay using human fibroblasts was performed to indirectly test the cytotoxicity of the PVA/amnion material, according to [38,39]. The MTT assay is a colorimetric assay for assessing cell metabolic activity and is commonly used when cytotoxicity of the polymer materials is considered, and also if the extract is used. The test is based on reducing the tetrazolium dye MTT 3-(4,5-dimethylthiazol-2-yl)-2,5-diphenyltetrazolium bromide to its insoluble formazan. The greater the formazan concentration, the deeper the purple color and, thus, the higher the absorbance indicating the high number of viable cells.

**Figure 6:**
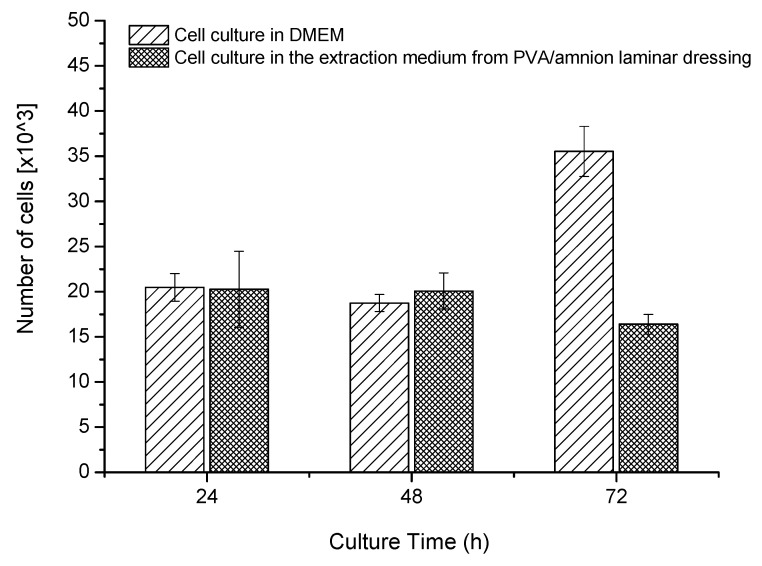
MTT test results for cells cultured in the presence of the extract from PVA/amnion laminar dressing.

Fibroblasts were cultivated for 1, 2, and 3 days with the extraction medium obtained from the PVA/amnion laminar dressing soaked in DMEM. The medium with cells was subjected to MTT studies. The results from the conducted studies are presented in Figure 6. For the first 48 h, the number of cells in both DMEM and in the extraction medium from the PVA/amnion laminar dressing were at the same level. After 72 h, the number of cells in the PVA/amnion extraction medium stayed at almost the same level, while cells in the DMEM control medium increased in number.

Fibroblasts were able to grow for 24 and 48 h of culture in the presence of extract of PVA/amnion laminar material. No exponential cell growth in extract was visible between 48 and 72 h, and additionally a decrease in the number of cells in comparison to the DMEM control was observed. This result suggests that at this stage of culture, the biomaterial exhibits cytostatic or toxic effects. Based on these results, we assume that it is possible to apply the fabricated laminar dressing for wound healing, but only for a shorter time. In clinical practice, such short-term dressings are very often used. These preliminary results are promising, however, we are aware that direct cytotoxicity tests are necessary to confirm the applicability of the obtained material, what is forthcoming.

#### 3.3.2. Cell Proliferation and Death Studies

The test for proliferation and cell death was performed to evaluate the in vitro performance of the dressing on the fibroblast culture. Human fibroblasts were grown in the extract from the PVA/amnion laminar dressing for 24 and 72 h. After an appropriate time, cells were detached with trypsin solution and stained using the Tali™ Apoptosis Assay Kit with Annexin V-Alexa Fluor™ 488 and Propidium Iodide. The apoptotic cells display green fluorescence, dead cells display red and green fluorescence, and live cells show little or no fluorescence. The presented method of proliferation and apoptosis testing is not a method included in the ISO norm standard. The authors’ intention is to show its potential, in the determination of the interaction of extract from polymer material with cells, which after an appropriate validation procedure can become a comparative test, for example, to the MTT assay.

**Figure 7:**
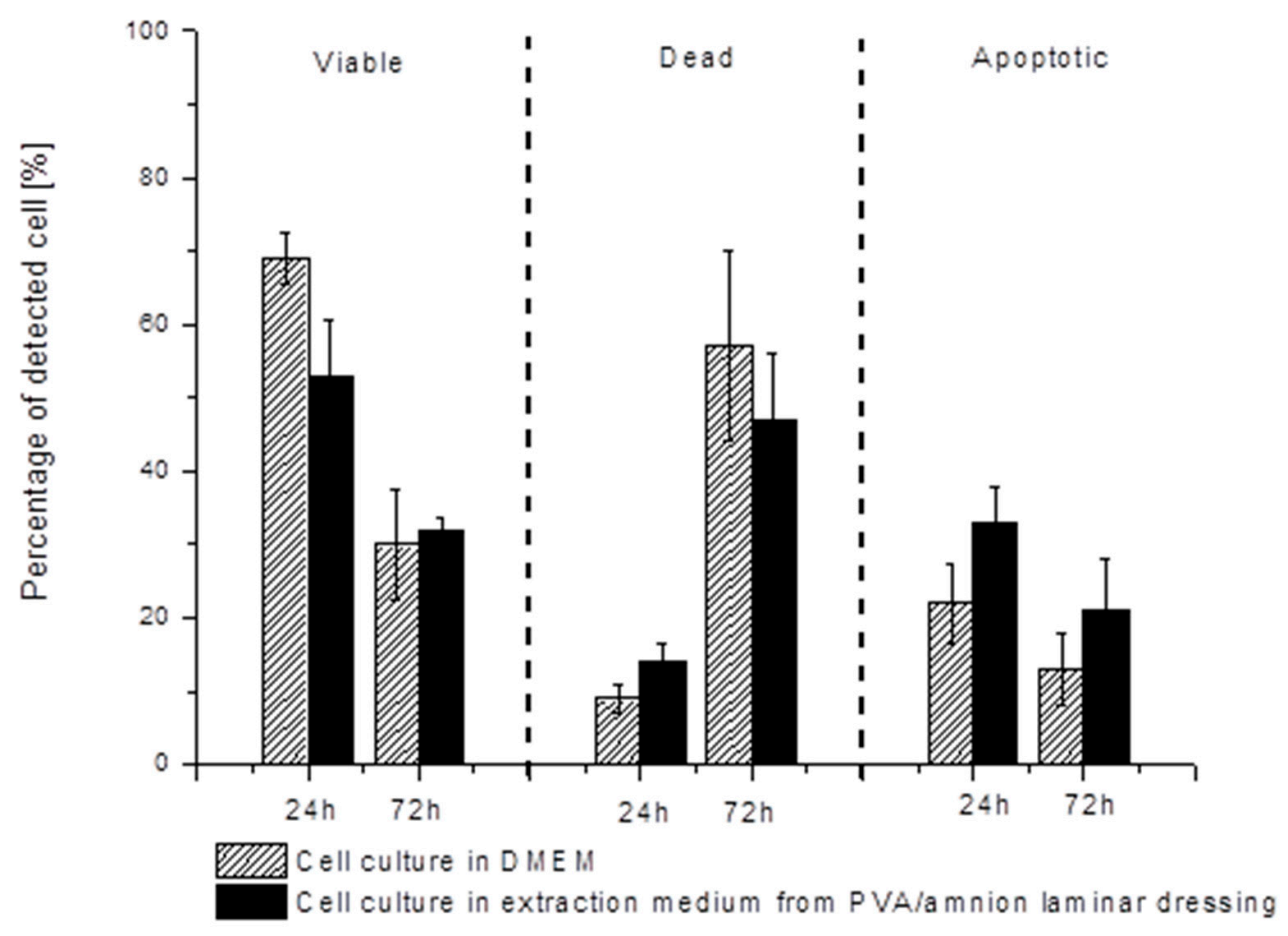
Viable, dead, and apoptotic cells detected during the fibroblast culture in the extraction medium from PVA/amnion laminar dressing.

The results of the performed test are presented in Figure 7 as a percentage of the viable, dead, and apoptotic cells versus culture time.

According to the obtained results, the percentage of the viable, dead, and apoptotic cells after 24 h and 72 h of cell culture did not differ significantly as compared to the control. After 24 h of cell culture, a certain percentage of dead and apoptotic cells could be detected. The amount of these cells increased as the cell culture time increased. This preliminary research indicates that the PVA/amnion laminated dressing has no significant detrimental effect on skin cells, mainly for the shorter culture times. Further biological studies of cells cultured in the presence of PVA/amnion laminar dressing, e.g., genotoxicity, are obligatory and forthcoming, but in this study, our intention was to show the potential of the obtained dressing for wound healing, at least as a short-term material.

#### 3.3.3. Antibacterial Studies

**Figure 8:**
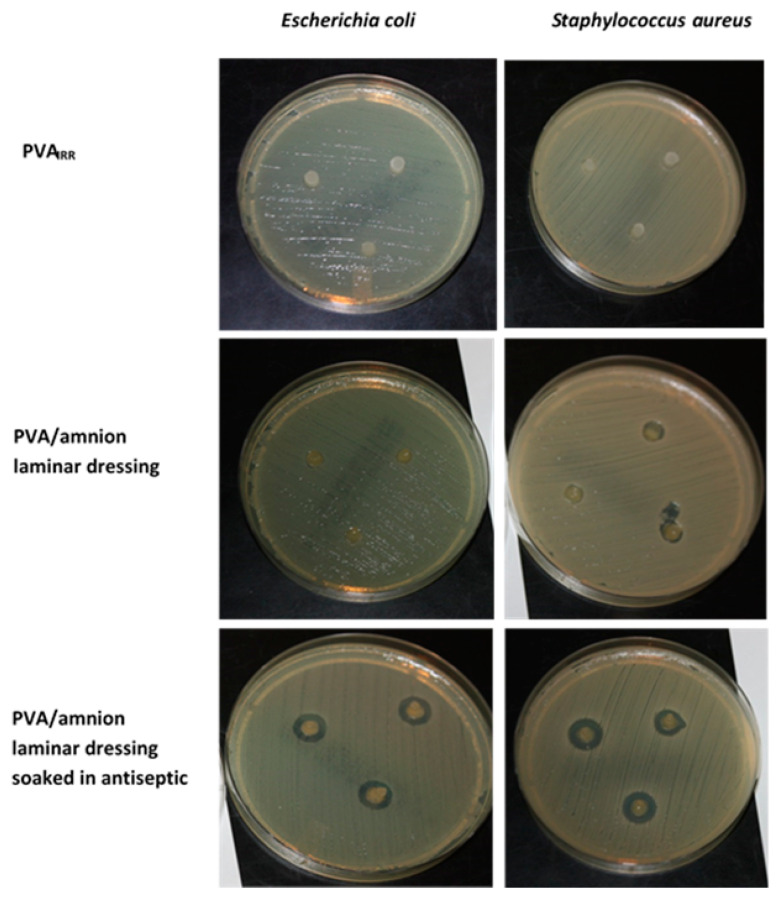
Antimicrobial activity of the cryogels evaluated using a disk diffusion method performed with *S. aureus* and *E. coli*.

The antibacterial activity of the laminar dressing was investigated through inhibition zones. As the cryogel and the amnion itself possess absorbent properties, they can be used as a carrier of, for example, antiseptics for the treatment of infected wounds. Based on our own experience, as an antiseptic, we have chosen Sutrisept. Soaking the PVA/amnion laminar dressing in the antiseptic did not change its structure and morphology for one week at least (data not shown). The antibacterial activity of the dressings was studied for two strains of bacteria most often isolated from infected patients` burn wounds: the Gram-positive strain *Staphylococcus aureus* and the Gram-negative strain *Escherichia coli*. The obtained results are presented in Figure 8. The PVA/amnion dressings showed great absorption properties when soaked with the antiseptic. Such a prepared dressing inhibited bacterial growth. Based on the literature data [55], our clinical experience, and the studies presented in this article considering the growth inhibition zone, it can be concluded that the obtained PVA/amnion laminar dressing can prevent infections when used on uncontaminated wounds. In the case of infected wounds, the dressing can be soaked with antiseptic, leading to the efficient inhibition of bacterial growth.

#### 3.3.4. Histological Examinations of the Dressing with the Cells

**Figure 9:**
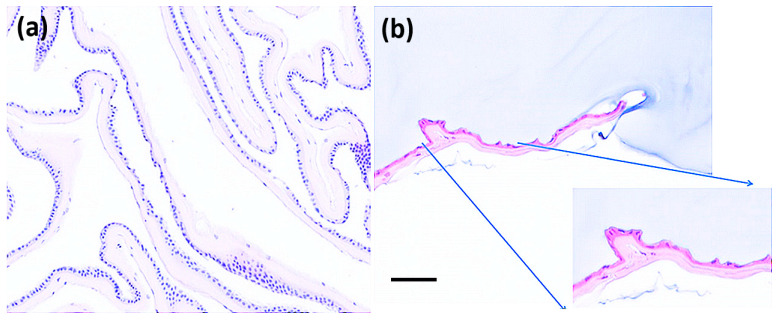
Results of (**a**) histological examination of amnion (magnification 10×) and (**b**) laminar dressing composed of amnion grafted to the cryogel (magnification 20×, space bar 200 μm; PVA_IRR_ 10 wt%, 35 kGy).

The results of the histological studies on the amnion and amnion attached to the cryogel are presented in Figure 9. Microscopic evaluation of the histological structure of the amnion showed clearly the epithelial, basement, compact, and fibroblast layers. The spongy layer was difficult to discern. The histological examination of the laminar dressing showed that the amnion was attached to the cryogel from the side of the epithelial cells. There is visible partial detachment of the amnion from the cryogel surface, probably due to the inaccurate overlaying of the amnion leading to the formation of air bubbles.

The histological observation confirms that the presence of PVA cryogel does not disturb the structure of the amnion. The histopathological similarity between the amnion and the skin indicates that the obtained PVA/amnion laminar dressing constitutes a promising material for the wound healing process.

## 4. Conclusions

In the presented article, an easy preparation of the amnion-based laminar dressing is presented. The steps during the formation of the dressing are minimized as much as possible, as the sterilization of the dressing with an e-beam is performed simultaneously with its preparation. The layer made of PVA cryogel serves as the carrier of the amnion coating. As a result of this approach, the amnion can be easily applied to the wound, and the entire dressing can be easily manipulated. The shape of the dressing can be simply selected by using an appropriate mold for pouring the gel. In addition, the gel can be an excellent reservoir of medical products, e.g., antiseptics, which further extends the possibility of using the obtained PVA/amnion laminar dressings. Based on the performed biological experiments, we have shown that the obtained dressing does not have a harmful effect on skin cells, mainly for shorter culture times. For longer culture times, it exhibited cytostatic properties. The histological examination revealed that the structure of the amnion attached to the cryogel was preserved. The preliminary results of biological research obtained in this study pointed out that the obtained amnion-based laminar dressing is a promising alternative to the amnion itself used as a dressing for wound healing. It combines the properties of the amnion with the properties of a hydrogel dressing, such as providing a moist environment, the possibility of encapsulating biologically active substances, and easy preparation and manipulation during application.

## Figures and Tables

**Table 1:** The characteristics of the PVA cryogels before and after e-beam irradiation.

	Sample	Temperature ^a^	GF [%]	Equilibrium SD [%]
**PVA_NOT-IRR_**	PVA 5%	40 °C	81	505
		90 °C	disintegration	
	PVA 10%	40 °C	96	325
		90 °C	disintegration	
**PVA_IRR_**	PVA 5% 25 kGy	40 °C	96 ± 1	510 ± 2
	PVA 5% 35 kGy		97 ± 1	506 ± 3
	PVA 10% 25 kGy		96 ± 1	348 ± 4
	PVA 10% 35 kGy		96 ± 1	357 ± 7
	PVA 5% 25 kGy	90 °C	90 ± 1	310 ± 3
	PVA 5% 35 kGy		91 ± 1	334 ± 3
	PVA 10% 25 kGy		89 ± 1	247 ± 5
	PVA 10% 35 kGy		91 ± 1	242 ± 8

^a^ temperature at which the samples were washed out before *GF* and *SD* measurements.

**Table 2:** The relative crystallinity of PVA cryogels.

Relative Crystallinity, %	5% PVA	10% PVA
**PVA_NOT-IRR_**	0.69 ± 0.53	2.16 ± 1.43
**PVA_IRR_**	0.61 ± 0.55	2.12 ± 1.37

**Table 3:** Mechanical properties of studied PVA_IRR_ cryogels.

Sample	Shore Hardness	Young Modulus [KPa]
**5% PVA 25 kGy**	0.15 ± 0.02	161
**5% PVA 35 kGy**	0.75 ± 0.05	171
**10% PVA 25 kGy**	2.95 ± 0.11	227
**10% PVA 35 kGy**	4.50 ± 0.10	266

## Data Availability

The data presented in this study are available on request from the corresponding author.
